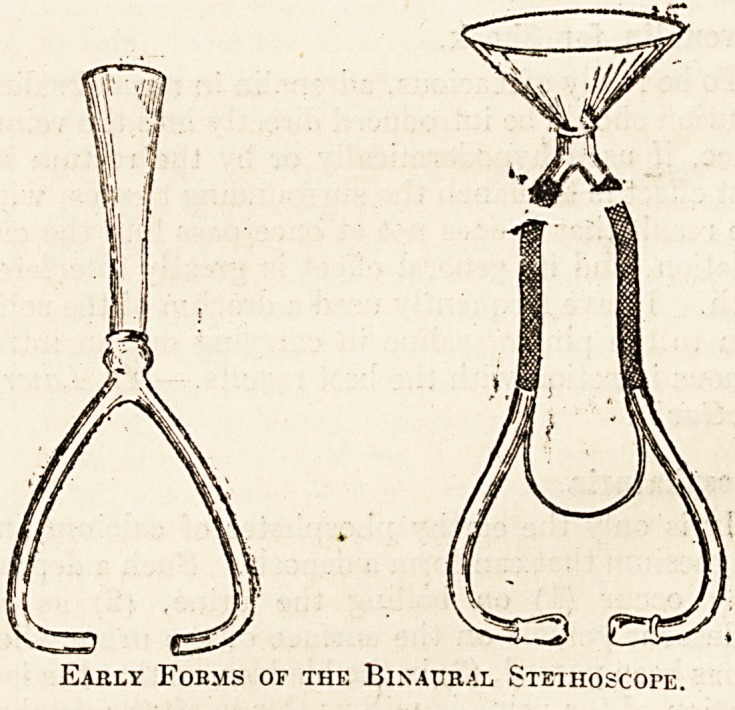# The Stethoscope

**Published:** 1910-12-24

**Authors:** 


### THE STETHOSCOPE.

### ITS HISTORY AND EVOLUTION.*

The practice of listening to the respiratory and other sounds in the thoracic or abdominal cavities of a patient is a very old one. "Let the physician train his eye, his ear, his nostril, his finger, and, above all, his eye," says Susruta in a treatise which experts date back to 3000 B.C., and it seems clear that the Indian physicians knew and practised the method known as "immediate auscultation." That Hippocrates knew it as well is proved by a passage in "De Morbis"; that others knew of it is equally certain. The famous lady doctor of Salern put her ear over "the swollen integument of the belly whereunder the morbid urine had become extravasated," and was thereby able to hear "creaking like the wind stirring dead leaves"—a singularly poetical description of so unpoetical a condition as surgical emphysema.

#### Immediate Auscultation.

But there is no evidence to prove that these observers made use of immediate auscultation as a regular aid to clinical diagnosis. They considered the passage in Hippocrates (as Laennec himself confesses he had considered it) "one of the mistakes of that great man, which it were a kindness to forget." Nature's secrets are sometimes more easily betrayed by chance than obtained by labour; and immediate auscultation, though mentioned in old text-books, was little practised, because, to quote the words of the learned Boerhaave, "its findings are oft inconclusive, subversive to true knowledge and fallacious."

#### The Invention of the Stethoscope.

In 1816 there came to the Hôpital Necker at Paris, as its chief physician, a "slight, rather wizened-looking individual. This individual was M. le docteur Laennec, a Breton gentleman, who could trace his line back to royalty, and whose heroic services during the campaign of 1814 had won him honourable recognition from the French Government. To the profession he was already known as an able pathologist, as a man who had not specialised in any branch of medicine, but had adopted the maxim of Averroes that "a physician ought to know everything of his art." He had written largely, and his articles, composed in a style far superior to that of other contemporary professional writings, had

Portrait of Laennec.

already attracted considerable notice and evoked some discussion. It was this wizened "old-looking young man" who had "discovered" encephaloid carcinoma and melanotic sarcoma; he, too, had done yeoman service at the Salpêtrière. It is true there were some who sneered at his claims. These critics pointed out that Hey in 1803, and Abernethy in 1805 (not to speak of Burns as early as 1800), had both described melanosis, and spoke with contempt of the young chief physician's merits. But Laennec disarmed all opposition by his quiet, unassuming

* For many of the facts mentioned in this article we are indebted to a paper read before the Polyclinic some years ago by Dr. Leonard Williams.

gentleness, his ready attention to criticism, and his zealous energy in his hospital duties. In that manner he obtained a fairly large private practice, and it is worthy to note that his discovery of the stethoscope was made in his private and not in his hospital practice.

"I was consulted," he writes, in relating his discovery, "by a young woman labouring under general cardiac disease. Percussion, pulvation, and palpation were of little avail in this case on account of the superficial fat. On account of the sex of the patient immediate auscultation was rendered inadmissible. I happened to recollect a simple fact in acoustics. This fact was the augmented impression of sound when conveyed to the ear through certain solid bodies—as when we hear the scratch of a pin drawn over a deal board to the other end of which we have applied our ear. I rolled a quire of paper into a kind of cylinder. I applied one end of this cylinder to the region of the hear; the other end I held to my ear. Judge of my surprise and pleasure when I found that by this simple method I could appreciate the heart's action much more clearly and distinctly than I had ever before been able to do with immediate auscultation. . . . From that moment I perceived that this method might enable us to ascertain the character not only of the action of the heart, but of every kind of sound produced by the thoracic viscera—the voice, the rattle, perhaps even the fluctuation of fluid extravasated into the pleura or pericardium."

### Improving the Instrument.

He did not stop here. He attempted to improve his instrument. Goldbeater's skin, cardboard, a piece of intestine inflated with air, and a solid length of wood were successively experimented with, and finally he fixed upon a cylinder of light wood, an inch and a half in diameter and a foot in length, perforated by a longitudinal bore three lines wide, and funnel shaped at the extremities. With this primitive instrument Laennec did most of his auscultatory work. It is true, like a sensible clinician, he did not neglect the other aids at his disposal. One has only to read his monumental work on the diseases of respiration to find how carefully he trained himself to a high pitch of perfection by using each method in its place and by laying no undue stress on any one particular method. Armed with his wooden stethoscope he heard and minutely described rhonchi, redux crepitation of pneumonia, sibilant and crepitant râles, the metallic tinkling produced by the voice, pectoriloquy and bronchophony heard over areas of solid lung, and last, but not least, the normal and abnormal heart sounds and the murmurs produced by pressure over the large arteries. In June 1818 he gave a summary of his conclusions to the profession in his "De l'Auscultation Médiate, ou Traité du Diagnostic des Maladies des Poumons et du Cœur, fondé principalement sur ce nouveau moyen d'exploration." But his work had been in excess of his strength. Always delicate, he found that he could not shake off a cold contracted in the autumn, and was obliged to resign his appointment at the Necker Hospital and return to his native town of Quimper, in Brittany.

### Laennec's Later Years.

Here he spent two years, living in seclusion in a little cottage which overlooked the waters of the bay of Douarnenez. He had a harassing cough, with little expectoration, but with frequent attacks of vertigo and fainting, and "a depression of spirits amounting almost to a tedium vitæ." It was the beginning of that disease of which he himself had written so much, and which was to prove fatal in his case. He does not appear to have been cognisant of its gravity. In his little shore cottage he experimented with his instrument, and received daily reports from his friends at Paris about the reception of his discovery by the profession. These reports were not encouraging. There was a disposition to scoff at the new method, and to call its usefulness into question. On the other hand, some of the leaders of the profession in France—and, be it also added, in England—were not slow to recognise the importance of Laennec's discovery, and his friends requested him to hasten back to Paris. He did so. He resumed his course of lectures at the Necker, whither the fame of the pathological clinician had already attracted large numbers of foreign students. On the death of M. Halle he was appointed to the Chair of Medicine in the College of France. Three years later his health became so gravely impaired that he had to retire to the country. He never returned to Paris, but died at Quimper on August 13, 1826, at the early age of forty-three.

How much the stethoscope has done for the advancement of our knowledge of pulmonary and cardiac disorders, anyone reading and comparing what has been written since the time of its discovery with what was stated about such common conditions as valvular disease, bronchitis, phthisis, and empyæma, will be able to see for himself. The new method introduced by Laennec promised much towards the simplification of diagnosis, and as much, perhaps, towards the betterment of existing modes of treatment. With the old aids, palpation and percussion, only a marked improvement or the reverse could be made out. With the more accurate method of the senior physician of the Necker Hospital a comparatively small change in the condition could be definitely diagnosed. But much had to be learnt as to the application of the new instrument. Professional men, accustomed to the old methods, sneered at the invention. They could hear nothing with it, they declared. Laennec's aphorisms were moonshine; his conclusions unreliable.

### Modifications of the Stethoscope.

Sir Charles Scuddemore, who went to Paris as soon as he heard of the new method, expressed himself in terms of indignant astonishment. "Why, I can't hear anything," he cried. Laennec saw where the difficulty lay; not in the instrument, but simply in the fact that Sir Charles possessed an unusually large tragus. So the first modification of the stethoscope was made specially for Sir Charles Scuddemore's benefit. It consisted in hollowing the ear-piece. Piory suggested a further improvement in removing the large mass of wood at the ends and leaving only a slightly concave ear-piece and a larger chest-piece. Dr. C. Williams added a further improvement by drawing out the ear-piece into a bell-shaped form, by which the sounds were intensified. The result of his suggestions was the adoption of the "trumpet-shaped stethoscope." Other experimenters modified the shape still further, while others, again, tried various materials in turn in the hope of getting a better conducting material. Old stethoscopes were made of mahogany or walnut; pear wood, owing to its lightness and porousness, was suggested, but seems never to have been adopted. Ebonite was a favourite with English physicians, owing to the readiness with which it could be cleaned.

### The Binaural Stethoscope.

A further modification, first suggested by an Edinburgh instrument maker, consisted in a solid

THE EVOLUTION OF THE SINGLE STETHOSCOPE.

chest-piece with an open tube communicating with it. The end of this tube fitted into the listener's ear. The suggestion was made on the ground that with an ordinary ear-trumpet, such as used by the deaf, the heart sounds could be heard. In this way the mon-aural stethoscope was invented. But it was soon found that the sounds heard by the tubal stethoscope were not so definite nor so clear as those to be heard when using the ordinary wooden instrument. The suggestion was made that an improvement in strength and audibility would follow the use of two ear-tubes instead of one. Dr. Williams, who as early as 1829 adopted a double ear-piece, must be given the credit of having invented the binaural stethoscope. Leared's modification, in many respects similar to the instruments now in general use, was put on the market in 1851, and its greater flexibility, together with the fact that the sounds which it transmitted were better intensified than with

THE FLEXIBLE STETHOSCOPE.

the single-stemmed instruments, made it a favourite. Since that time innumerable modifications have been placed on the market, and the fashion in stethoscopes is perhaps as diversified as that in hypodermic syringes or trusses.

### The Modern Instrument.

The modern clinical stethoscope is manufactured with extreme care and attention. Many models are on the market, some of most elaborate construction, being specially fitted with ivory or celluloid ear and

EARLY FORMS OF THE BINAURAL STETHOSCOPE.

chest-pieces, and with hardened rubber tubing for use in tropical climates. A comparatively recent model is the "Clinical Combination Stethoscope," which consists of a specially large chest-piece from which proceed several tubes fitted with ear-pieces. With this instrument a demonstrator can enable as many as three students to listen to the special sound over the patient's chest, without having to remove the chest-piece and ask each student to apply his own instrument. Whether multiple instruments of this kind are of any great value is open to question. In fact, there are some observers who hold that a binaural stethoscope itself is not an unmixed blessing to the diagnostician. It enhances the sound, it hardens a bruit, and it fails to detect murmurs of very low intensity. For that reason the experienced ascultator prefers to use the old wooden instrument in cases where there is a doubt as to the presence or absence of, say, a diastolic aortic bruit.

### The Value of Auscultation in the Diagnosis of Cardiac and Pulmonary Diseases.

The rules laid down by Laennec for the use of the stethoscope apply to-day, when we have better instruments and more experience to guide us in their application, as much as they did at the time when he drew them up. Laennec laid down three desiderata. "The stethoscope," he wrote, "must be applied very exactly and perpendicularly to the surface, so as to leave no interval between the skin and any part of the extremity applied; great care must be taken not to produce pain by too strong pressure; the examiner should be careful not to place himself in an uncomfortable position, and should not hold his head backwards." Laennec believed that he could auscultate as clearly and accurately through clothing; the only thing he stipulated was that the clothes should be "tightly applied" to the body of the patient. All observers, however, are now agreed that auscultation, except by applying the chest-piece direct to the uncovered skin, is liable to give fallacious impressions.

Nowadays the physician has many other aids to help him in differentiating between the various cardiac and pulmonary complaints, and it is possible that as these aids increase, the value of the stethoscope will become lessened. *X*-rays, bacteriological examinations, and graphic representations of the heart's action are all pressed into service in making a diagnosis. But the majority of these new helps to diagnosis are outside the limits of the general practitioner's time, patience, or means. For him the stethoscope must remain, as it was to the consultant in the days of Laennec, one of the greatest aids that are available for the diagnosis of pulmonary and cardiac disease.

## Figures and Tables

**Figure f1:**
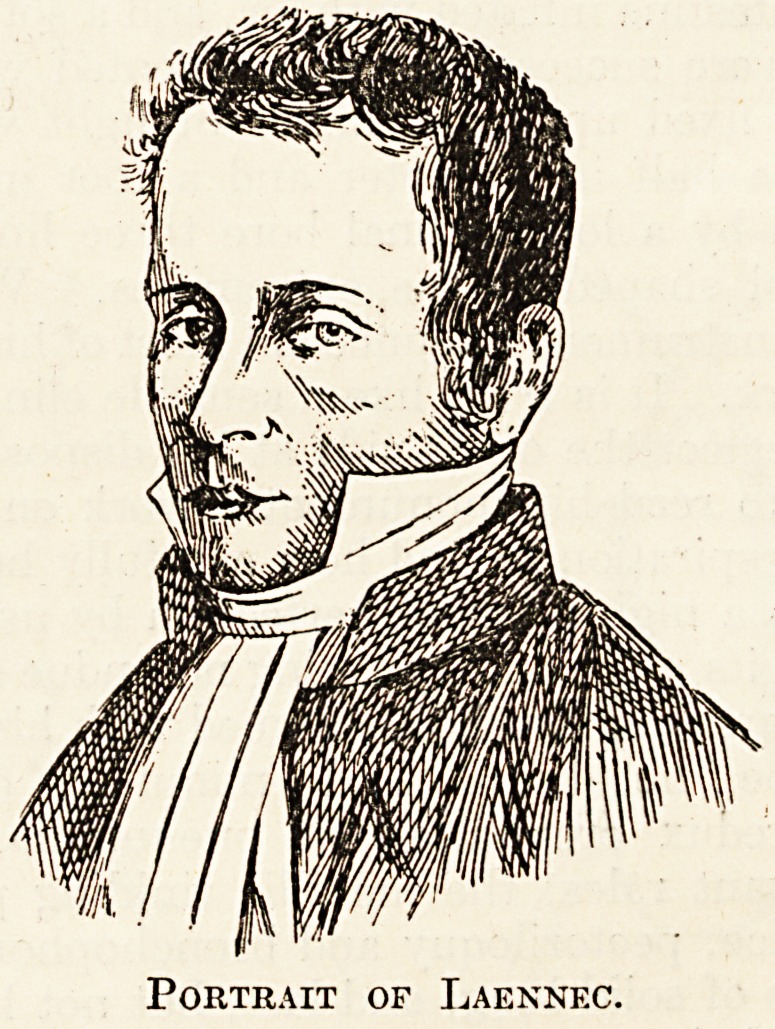


**Figure f2:**
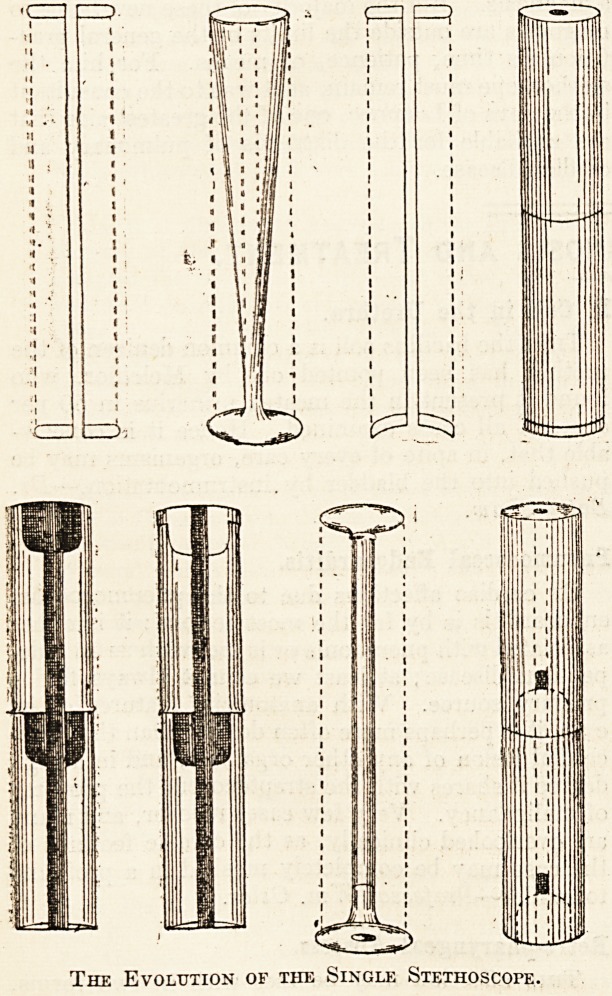


**Figure f3:**
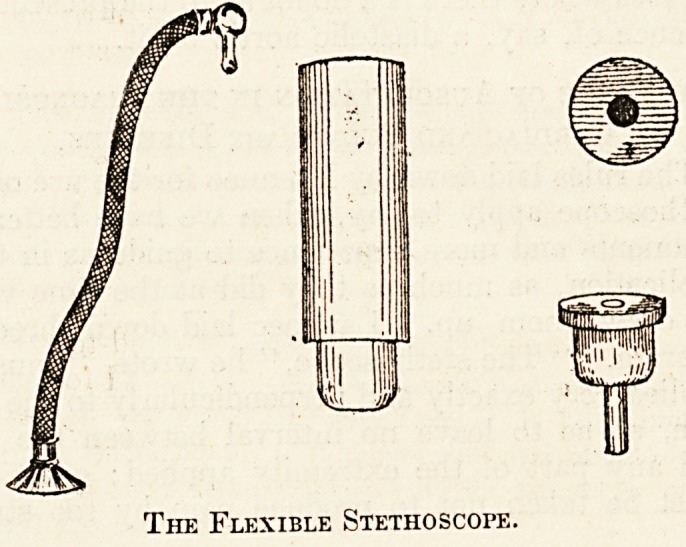


**Figure f4:**